# Imported Cutaneous Larva Migrans in an Adolescent Traveler: A Case Report From Chile

**DOI:** 10.7759/cureus.104834

**Published:** 2026-03-07

**Authors:** Diego Guarda, Cristóbal Norambuena, Priscilla Marquez, Gerardo Bascuñan, Diego I Mendez-Villanueva

**Affiliations:** 1 General Practice, Clínica Andes Salud, Puerto Montt, CHL; 2 General Practice, Universidad Andres Bello, Santiago, CHL; 3 General Practice, Hospital Familiar y Comunitario de Carahue, Carahue, CHL; 4 Dermatology, Universidad de Santiago de Chile, Santiago, CHL

**Keywords:** cutaneous larva migrans, ivermectin, serpiginous rash, travel-related dermatoses, zoonotic hookworm

## Abstract

Cutaneous larva migrans (CLM) is a parasitic dermatosis caused by the superficial migration of animal hookworm larvae in human skin, typically acquired through contact with contaminated soil or sand in tropical environments. We report the case of a 17-year-old female patient who presented in Chile with pruritic serpiginous skin lesions following recent travel to Brazil. Diagnosis was established clinically based on characteristic morphology and exposure history. The patient was treated with oral ivermectin, resulting in complete resolution of active lesions and symptoms. Given the rarity of locally reported cases in Chile, this report underscores the importance of clinical recognition in nonendemic settings to ensure prompt treatment and avoid unnecessary diagnostic procedures.

## Introduction

Cutaneous larva migrans (CLM), also known as creeping eruption, is a parasitic dermatosis caused by the superficial migration of nematode larvae within the epidermis, producing characteristic linear or serpiginous pruritic tracks [1]. In humans, CLM is most commonly associated with animal hookworms, particularly *Ancylostoma braziliense* and *Ancylostoma caninum*, with humans acting as accidental hosts in whom the parasite cannot complete its life cycle [2]. Infection typically occurs after direct skin contact with soil or sand contaminated with feces from infected dogs or cats, especially in warm and humid environments [1,2].

Globally, CLM is frequently encountered in tropical and subtropical regions and represents one of the most common travel-related parasitic dermatoses [3,4]. Its epidemiology is influenced by environmental contamination, animal reservoirs, and climatic conditions favoring larval survival [1,4]. In endemic communities, prevalence has been associated with socioeconomic and environmental risk factors [5], whereas in nonendemic countries, most cases are imported and diagnosed in returning travelers [3,4].

In Chile, CLM is uncommon, with only one autochthonous case formally reported in the national literature [6]. This epidemiological context makes recognition particularly relevant, as unfamiliarity may lead to diagnostic confusion with conditions such as cellulitis, contact dermatitis, scabies, or other inflammatory dermatoses. The present case highlights the importance of clinical suspicion in a nonendemic setting, especially in patients with recent travel exposure, to ensure timely diagnosis and appropriate treatment.

## Case presentation

In February 2026, a 17-year-old Chilean female patient with no relevant past medical history presented with pruritic skin lesions of five days’ duration. A key epidemiological factor was a recent school trip to Brazil, approximately two weeks prior to symptom onset. She denied recent use of new medications, changes in perfumes, cosmetics, or personal hygiene products, and had no history of contact dermatitis.

**Figure 1:**
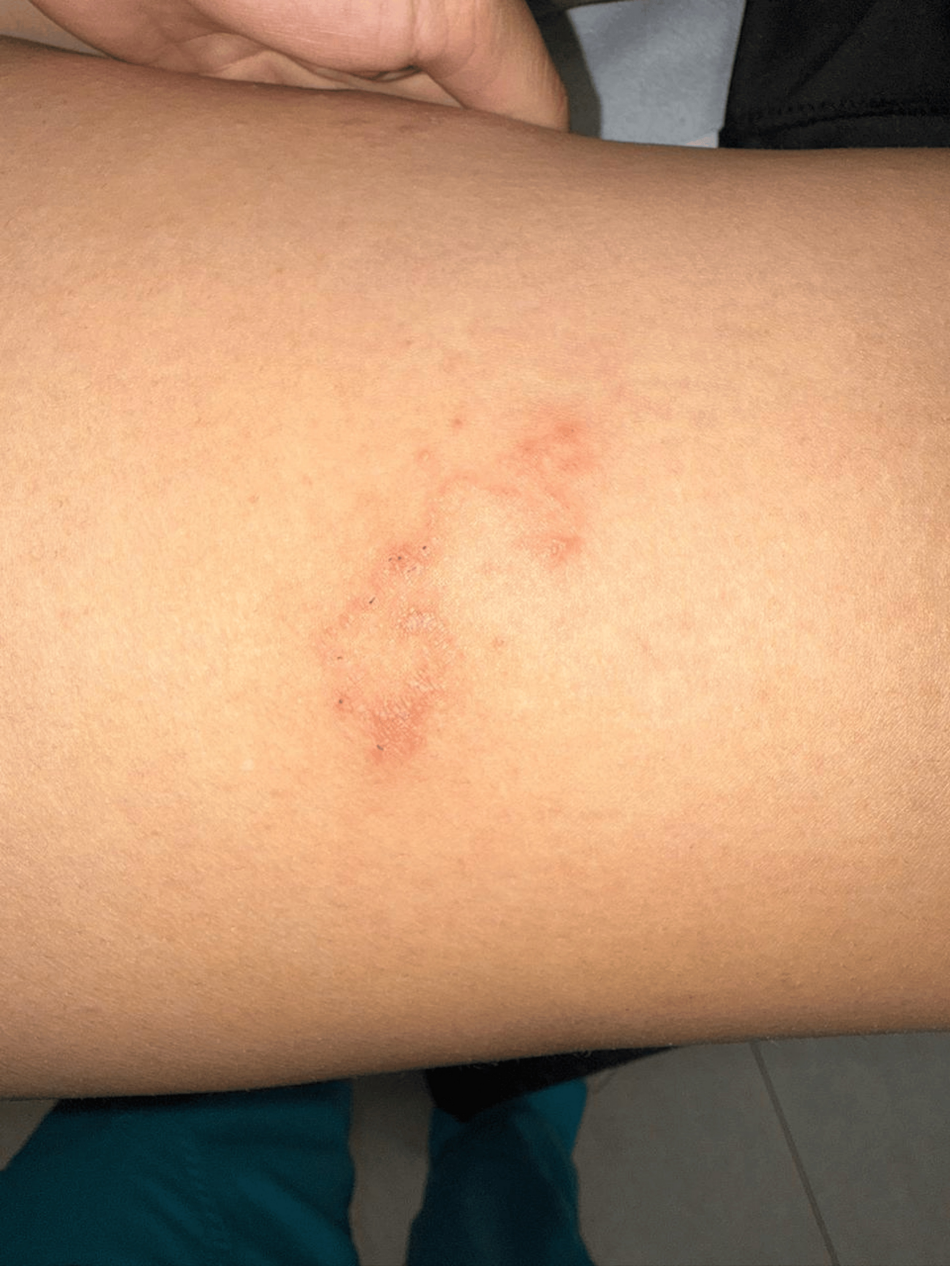
Cutaneous larva migrans on the anterior right thigh at presentation Linear and serpiginous erythematous lesion composed of papules and small vesicles on the anterior aspect of the right thigh, associated with excoriations secondary to scratching, consistent with active cutaneous larva migrans at initial presentation

The condition began as a pruritic wheal on the anterior aspect of the right thigh, which over subsequent days progressed into multiple papules and several vesicles that coalesced into a linear and serpiginous configuration, associated with excoriations secondary to scratching (Figure 1).

**Figure 2:**
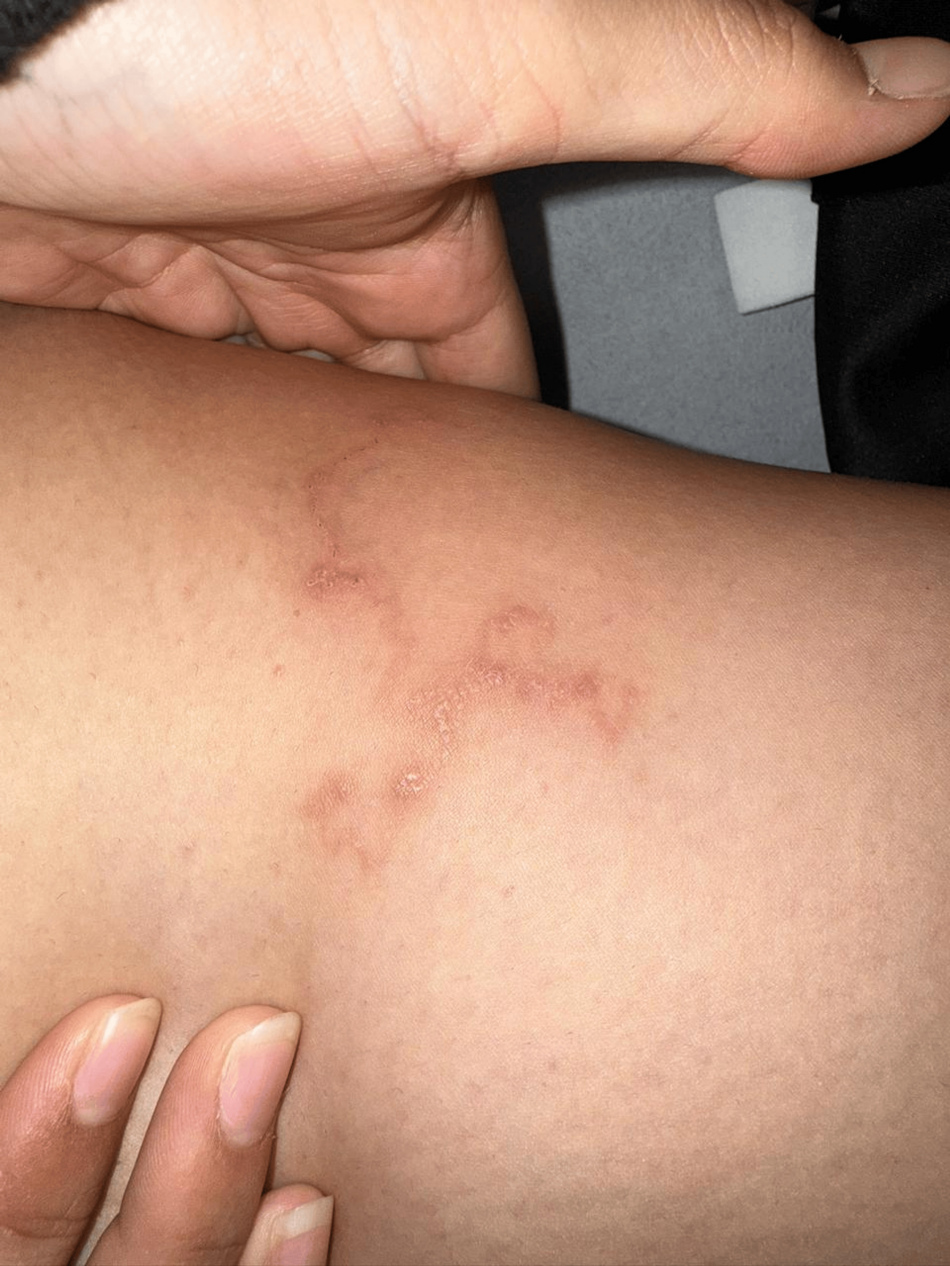
Serpiginous lesion on the medial aspect of the right thigh Additional serpiginous erythematous lesion with similar morphology involving the medial aspect of the right thigh at the time of diagnosis, supporting the clinical diagnosis of cutaneous larva migrans

On physical examination, an additional lesion with similar morphology was identified along the medial aspect of the right thigh (Figure 2). The patient was afebrile, in good general condition, and reported no systemic symptoms.

Laboratory studies showed a normal total leukocyte count (9,200 cells/µL) with mild eosinophilia (8%; absolute eosinophil count: 850 cells/µL). Total immunoglobulin E (IgE) was elevated (236.1 IU/mL). Laboratory results are summarized in Table 1. 

**Table 1:** Laboratory results at presentation WBC: white blood cell count; AEC: absolute eosinophil count; IgE: immunoglobulin E *Reference values may vary slightly by laboratory and age

Test	Result	Units	Reference values*
WBC	9,200	cells/µL	4,500-11,000
Eosinophils (relative)	8	%	0-5
AEC	850	cells/µL	0-500
Total IgE	236.1	IU/mL	0-100

**Figure 3:**
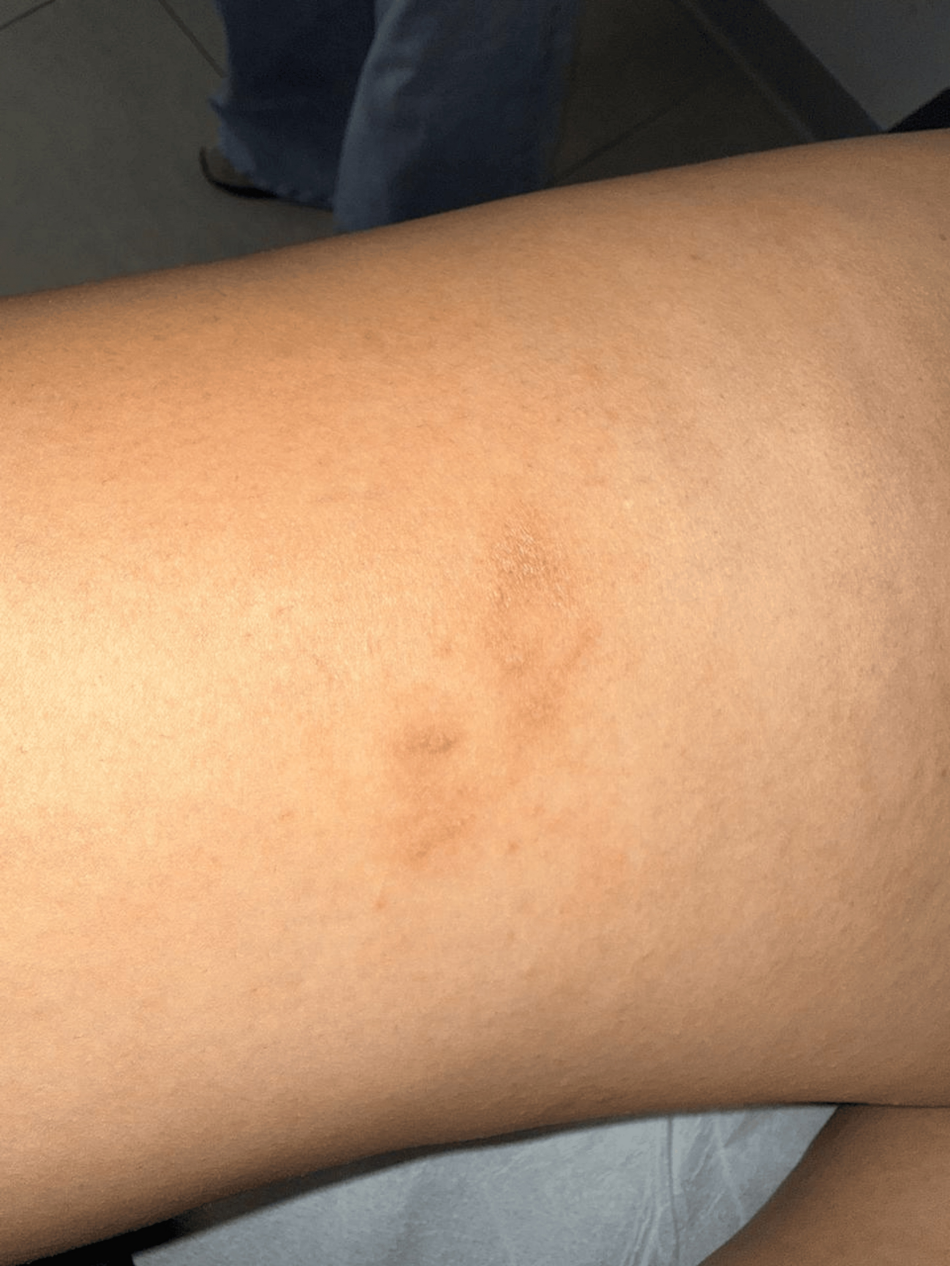
Posttreatment appearance of the anterior right thigh Follow-up image obtained three weeks after treatment with oral ivermectin showing complete resolution of active lesions, with mild residual scaling and postinflammatory hyperpigmentation on the anterior aspect of the right thigh

**Figure 4:**
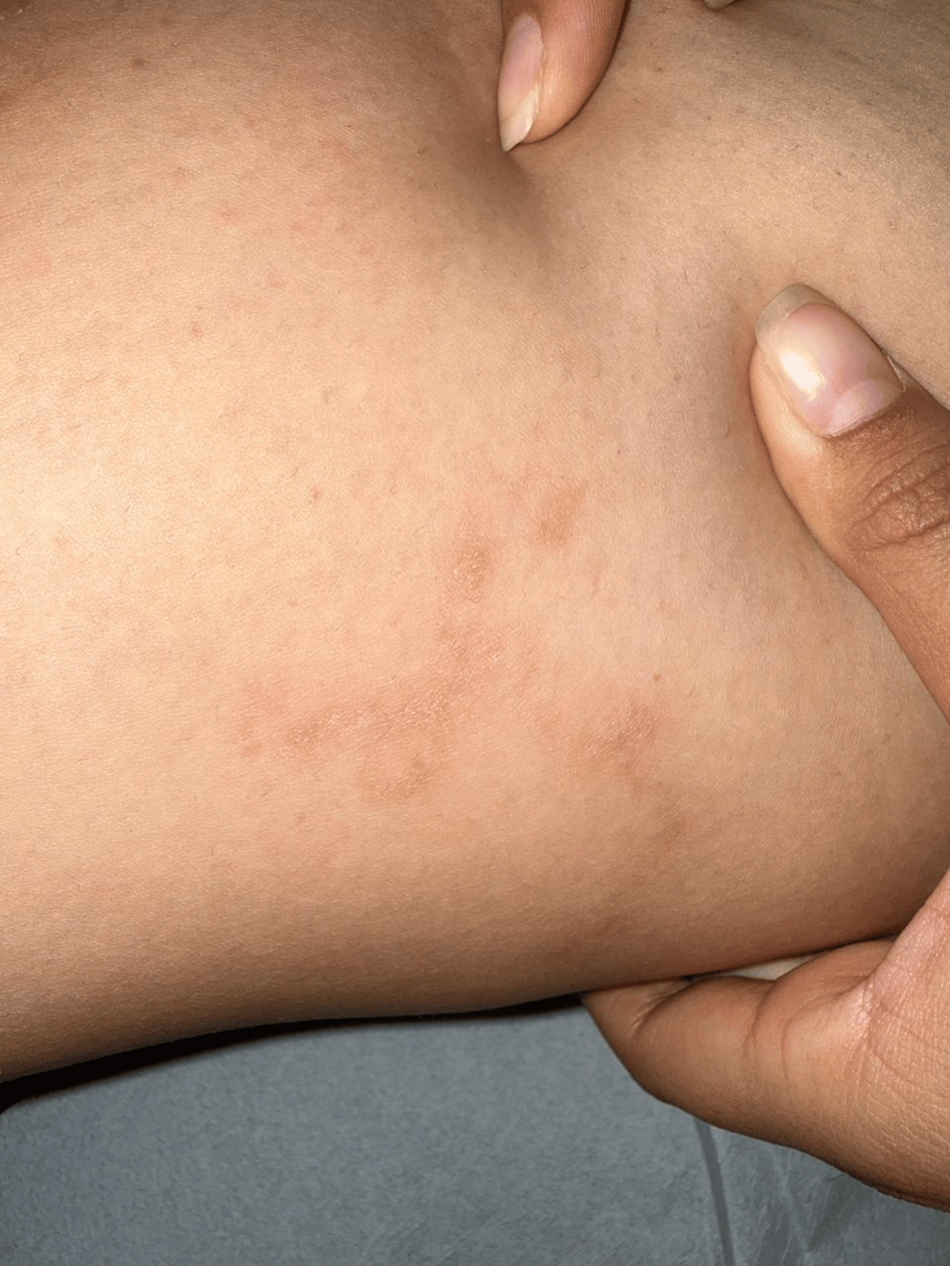
Posttreatment appearance of the medial right thigh Follow-up image of the medial aspect of the right thigh demonstrating absence of active serpiginous lesions, with residual postinflammatory hyperpigmentation after ivermectin treatment

Based on the characteristic morphology of the lesions, the compatible travel history, and laboratory findings, a clinical diagnosis of CLM was established. The patient was treated with oral ivermectin at a dose of 200 µg/kg as a single dose, with clinical follow-up scheduled three weeks later. At follow-up, she demonstrated an excellent clinical response, with complete resolution of pruritus and disappearance of active lesions. Residual findings consisted only of mild scaling and postinflammatory hyperpigmentation on both the anterior (Figure 3) and medial aspects of the right thigh (Figure 4). No adverse effects related to treatment were reported.

## Discussion

CLM results from skin penetration by infective filariform larvae of animal hookworms, followed by superficial intraepidermal migration [1,2]. The most commonly implicated species are *Ancylostoma* *braziliense* and *Ancylostoma ​​​​caninum *[2]. Because larvae are unable to penetrate the basement membrane in humans, the infestation remains confined to superficial skin layers and is ultimately self-limited, although it may persist for weeks and cause intense pruritus [1].

In endemic regions, CLM is associated with environmental contamination by animal feces and frequent barefoot contact with moist soil or sand [1,2]. Among travelers, CLM is one of the most frequently reported travel-related dermatoses, particularly following beach exposure in tropical destinations [3,4]. In endemic communities, prevalence and risk correlate with environmental exposure and socioeconomic conditions [5]. These patterns explain why most cases diagnosed in nonendemic countries are imported [3,4]. In Chile, local transmission has been exceptionally reported, with only one autochthonous case documented in the literature [6].

In nonendemic settings such as Chile, limited familiarity with this condition may lead to misdiagnosis as cellulitis, contact dermatitis, scabies, or other inflammatory dermatoses. Such diagnostic confusion can result in unnecessary laboratory investigations, imaging studies, or inappropriate antibiotic therapy. Early clinical recognition based on characteristic morphology and exposure history allows prompt treatment and prevents avoidable diagnostic procedures.

Recognized risk factors include walking barefoot, lying directly on sand, recreational beach activities, occupational exposure to moist soil, and contact with dogs or cats lacking routine antiparasitic control [1,2,6]. Secondary bacterial infection may occur due to excoriations from scratching [1].

CLM classically presents as a slowly advancing, erythematous, linear or serpiginous pruritic track [1,2]. Common sites include the feet, thighs, buttocks, and other areas in contact with contaminated soil [2]. Diagnosis is primarily clinical, supported by typical morphology and exposure history; direct identification of larvae is rarely feasible [2]. Differential diagnoses include *Strongyloides stercoralis *larva currens, tinea corporis, contact dermatitis, scabies, cellulitis, and arthropod bite reactions [1,2].

Although CLM may resolve spontaneously, treatment is recommended to shorten disease duration and relieve symptoms [1]. Oral ivermectin (200 µg/kg as a single dose) is widely used as first-line therapy, with high response rates [7-9]. Albendazole (400 mg daily for 3-7 days) represents an effective alternative [8-10]. Preventive strategies include wearing footwear, avoiding direct contact with sand or soil, traveler education, and routine deworming of pets [1,2,6].

## Conclusions

CLM should be considered in patients presenting with pruritic serpiginous skin lesions and a history of travel or exposure to contaminated soil or sand. In Chile, most cases are imported, and autochthonous transmission has been exceptionally reported. Clinical diagnosis allows prompt and effective treatment with ivermectin, preventing unnecessary investigations and improving patient outcomes.
